# *Stenotrophomonas maltophilia* of clinical origin display higher temperature tolerance comparing with environmental isolates

**DOI:** 10.1080/21505594.2025.2498669

**Published:** 2025-05-02

**Authors:** Laurita Klimkaitė, Radvilė Drevinskaitė, Karolis Krinickis, Edita Sužiedėlienė, Julija Armalytė

**Affiliations:** Institute of Biosciences, Life Sciences Center, Vilnius University, Vilnius, Lithuania

**Keywords:** *Stenotrophomonas maltophilia*, opportunistic pathogen, genotyping, virulence-related traits, antibiotic resistance, host body temperature

## Abstract

*Stenotrophomonas maltophilia* is a gram-negative, multidrug-resistant, opportunistic human pathogen responsible for hard-to-treat infections in immunocompromised individuals. Besides being recognized as an important clinical pathogen, *S. maltophilia* is also widespread in the natural environment, with knowledge of the pathogenic potential of the environmental *S. maltophilia* pool still lacking. In this study, we aimed to identify the differences in virulence-related traits between clinical and environmental *S. maltophilia* isolates by assessing their genotypic and phenotypic features. For this purpose, 40 *S. maltophilia* isolates from natural environment and 34 clinical isolates obtained from patients were analysed. We observed a high degree of genotypic diversity among the isolates irrespective of their origin. Although antibiotic resistance- and virulence-related genes were more prevalent in the clinical isolates, the majority of the analysed genes were also present in the environmental isolates. Most importantly, the phenotypic features, specifically the ability to form biofilms and display twitching motility at human body temperature were predominantly characteristic to the clinical isolates. Our study indicates that adaptation to endure human body temperature is a feature strongly linked to *S. maltophilia* strains of clinical origin, and is significant when differentiating harmless environmental bacteria from pathogenic *S. maltophilia* isolates.

## Introduction

*Stenotrophomonas maltophilia* is a gram-negative multidrug-resistant bacterium ubiquitously found in natural environments such as soil (most commonly in the plant rhizosphere), water (wastewater, ponds, river, and sea water), animal (mammals, fish) guts, and even everyday food products (dairy, meat, fresh vegetables) [1–5]. In recent years *S. maltophilia* has gained interest as an opportunistic human pathogen that causes hard-to-treat infections in immunocompromised patients [1].

As a pathogen, *S. maltophilia* displays a variety of virulence mechanisms related to its ability to survive in the human host and cause and progress infection [6]. *S. maltophilia* displays intrinsic resistance to a number of antibiotics and can acquire new resistance mechanisms via horizontal gene transfer and mutations [7]. *S. maltophilia* has been shown to possess cell-associated virulence-related structures (lipopolysaccharide, pili, non-pilus adhesins, flagella), as well as numerous extracellular virulence factors (extracellular enzymes (proteases, lipases, esterases, deoxyribonuclease, ribonuclease, hyaluronidase), haemolysins, cytotoxins, and siderophores) [8,9]. Biofilm formation is considered one of the major *S. maltophilia* trait allowing it to survive on both biotic and abiotic surfaces, escape from host defence mechanisms, and avoid the effects of antimicrobial agents [10]. Twitching motility displayed by *S. maltophilia* is another important virulence factor that helps pathogen to colonize biotic or abiotic surfaces and spread across them [11].

Besides being recognized as an important clinical pathogen, *S. maltophilia* is also known for its great biotechnological potential in bioremediation and plant biocontrol [12–14]. Therefore, it is important to understand and distinguish between the defining traits of virulent and harmless *S. maltophilia* variants. Studies analysing genotypic and phenotypic characteristics have been performed on clinical and environmental *S. maltophilia* isolates to differentiate between pathogenic and non-pathogenic strains. However, a deeper analysis of *S. maltophilia* revealed its high genotypic and phenotypic diversity [15–17]. In addition, clinical and environmental isolates have been shown to possess a similar distribution of virulence and antibiotic resistance genes, as well as phenotypic resistance to clinically relevant antibiotics, thus impeding the discrimination of *S. maltophilia* isolates according to their pathogenic potential [15].

The ability to grow and express virulence traits at human body temperature is an important feature that opportunistic pathogens have to possess [18]. Of the *Stenotrophomonas* genus, *S. maltophilia* is able to grow at 37°C, whereas its closest relative *Stenotrophomonas rhizophila* lacks this ability [19]. The ability of *S. maltophilia* to grow at 37°C has already been suggested as a very simple method differentiating between pathogenic and non-pathogenic isolates [19]. However, this observation was made by analysing only three *S. maltophilia* isolates of various origins [19], and the impact of temperature on the ability of environmental *S. maltophilia* to express virulence traits remains underexplored.

To address this issue, we aimed to identify differences in virulence-related traits between pathogenic and harmless *S. maltophilia*, looking into both phenotypic and genotypic features related to virulence. We showed that both clinical and environmental isolates harboured the majority of the analysed virulence-associated genes and were highly resistant to the tested antibiotics. However, the ability to express phenotypic virulence-associated features (form biofilms and use twitching motility) at the host body temperature were more characteristic to clinical *S. maltophilia* isolates. Here we propose that the adjustment to survival in the host body temperature could be the key adaptation for establishing the virulence of pathogenic *S. maltophilia* isolates.

## Methods

### Bacteria used in this study

The clinical and environmental *S. maltophilia* used in this study are listed in Supplementary Table S1.

### Collection of environmental *S. maltophilia* isolates

**Figure 1.**
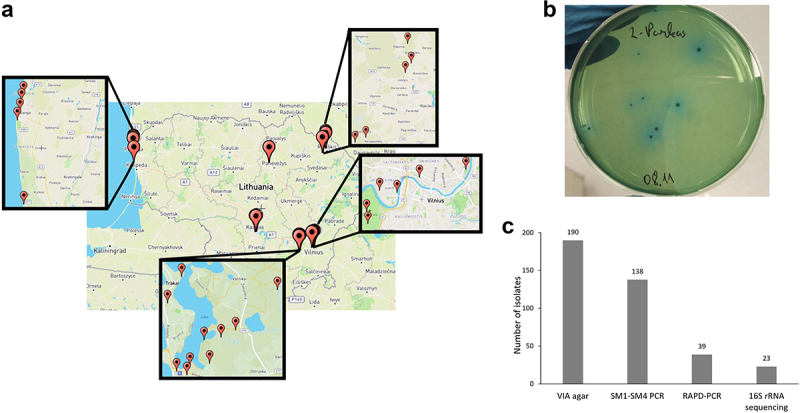
The isolation of *S. maltophilia* of environmental origin. (a.) Location of soil collection spots in Lithuania; (b.) Mannitol fermentation-positive isolates selection on VIA medium; c. Environmental *S. maltophilia* isolation outcome after each selection step.

For environmental *S. maltophilia* extraction, soil samples were collected from various parts of Lithuania during June–September 2021 (Figure 1(a)). A distance of at least 1 km was maintained between soil collection spots. In order to reach rhizosphere all soil samples were collected from 10 cm depth, after collection samples were stored at 4°C for up to 48 h.

For *S. maltophilia* isolation, soil samples (0.5 g) were mixed with 1 ml phosphate-buffered saline (PBS) and vortexed for 75 s (pulsing mode). Larger particles were removed by brief centrifugation, and supernatants were plated on selective medium containing vancomycin, imipenem, and amphotericin B (VIA medium) supplemented with mannitol/bromothymol blue indicator system [20]. Plates were incubated at 28°C for 72 h, mannitol fermentation positive colonies were further analysed. For initial genus identification, *Stenotrophomonas* specific primers SM1 and SM4 were used [21] (primers used in this study are presented in Supplementary Table S2). Isolates confirmed to belong to *Stenotrophomonas* genus were analysed by random amplified polymorphic DNA (RAPD) polymerase chain reaction (PCR) using OPA-02 and 380–7 primers to select unique isolates. The final assignment of *S. maltophilia* was performed using 16S rRNA gene sequencing (27F and 1492 R primers were used for 16S rRNA gene amplification, and the 515 R primer was used for Sanger sequencing reaction). Basic local alignment search tool (BLAST) was used to compare obtained sequences the National Center for Biotechnology Information (NCBI) data, *S. maltophilia* species were assigned if best search match was *S. maltophilia* with identity of 99% or more [22].

### Genotyping

Clinical and environmental *S. maltophilia* isolates were genotyped using BOX-PCR [16]. Briefly, DreamTaq Green PCR Master Mix (2X) (Thermo Fisher Scientific) with BOXA1R primers was used according to the manufacturer’s recommendations. The PCR cycles were set as follows: initial denaturation at 95°C for 3 min; 35 cycles of denaturation at 95°C for 30 s, primer annealing at 40°C for 30 s, and extension at 72°C for 8 min were performed; final extension was done at 72°C for 15 min. DNA samples were analysed by gel electrophoresis in 2% agarose with 1× TBE buffer. Gel band analysis was performed using GelCompar II software (Applied Maths), Dice coefficient was set at 1% with band tolerance set at 1.5% using the UPGMA method.

### Determination of antibiotic resistance and virulence-related genes

Standard polymerase chain reaction was performed to detect antibiotic resistance and virulence-related genes. DreamTaq Green PCR Master Mix (2X)(Thermo Fisher Scientific) was used according to the manufacturer’s recommendations. The primers used are listed in Supplementary Table S2. For integron gene cassette analysis, gene amplification was performed using Phusion™ Plus PCR Master Mix (Thermo Fisher Scientific), and amplified fragments were sequenced using the Sanger method at GENEWIZ (Azenta Life Sciences).

### Antibiotic susceptibility testing

Antimicrobial susceptibility testing of *S. maltophilia* isolates was performed using standard EUCAST disk diffusion testing method [23], susceptibility to trimethoprim-sulfamethoxazole (SXT), ciprofloxacin (CIP), tigecycline (TGC), ceftazidime (CAZ), gentamicin (GEN), and chloramphenicol (CHL) was evaluated. Results were interpreted according to the Clinical and Laboratory Standards Institute 2020 (CLSI-2020) breakpoints. At least three independent experiments were performed to evaluate resistance of each isolate.

### Growth measurements

To assess the ability of *S. maltophilia* isolates to grow in environmental (28°C) and host body (37°C) conditions, single colonies of isolates were inoculated into Tryptic Soy Broth (TSB) medium and grown for 20 h at 28°C temperature. Overnight cultures were diluted 50 fold, and the growth rate of the isolates was measured by Tecan Infinite M200 Pro plate reader as optical density at 600 nm (OD_600_) at 28°C or 37°C for 24 h (with shaking). Three independent experiments were performed to assess the growth of each isolate at both the environmental and host body temperatures.

### Biofilm formation assay

The biofilm-forming ability of environmental and clinical *S. maltophilia* isolates was evaluated using a crystal violet dye assay [24]. Briefly, overnight cultures were grown in TSB medium at 28°C for 20 h. The overnight cultures were diluted 50 fold and incubated in U-form shaped 96-well polystyrene plates (Nerbe Plus, Germany) at 28°C or 37°C temperature for 24 h. After the 24 h, the OD_600_ of the planktonic culture was measured, the wells were then washed with PBS buffer three times, and the biofilm formed was stained with 0.1% crystal violet solution for 15 min. Residual dye was removed with PBS buffer washing for five times, and the biofilm-bound crystal violet was dissolved in 200 μl of 99.6% ethanol per well, followed by OD_580_ measurement. The biofilm formation capacity was expressed as OD_580_/OD_600_. At least three independent experiments were performed to assess the ability to form biofilms by all the isolates at 28°C and 37°C temperature. Biofilm formation capacity was classified according to the cut-off value (ODc), which was expressed as OD_580_/OD_600_ of the mean negative control  +  3 × standard deviations. Four categories were used: non-producer (OD_580_/OD_600_ ≤ ODc), weak-producer [ODc < OD_580_/OD_600_ ≤2 × ODc], moderate-producer [2 × ODc < OD_580_/OD_600_ ≤4 × ODc], and strong-producer (OD > 4 × ODc) [25].

### Twitching motility assay

*S. maltophilia* twitching motility was evaluated using Macroscopic Twitching Assay on a semi-solid medium in a humid environment [26]. Briefly, 15 ml of freshly prepared 1% TSB medium with 1% agar was poured into petri plates, the plates were dried for 2 h in a laminar flow cabinet. One colony was selected using a sterile toothpick and stabbed through the agar, reaching the bottom of the Petri plate. The plates were incubated at 28°C or 37°C temperature for 48 h in humid airtight containers. Motility was evaluated by measuring the twitching motility halo (cm^2^) formed at the plastic-agar medium interface. Twitching motility levels were categorized similarly to biofilm formation using the negative control cut-off value ODc (mean area (cm^2^) of negative control  +  3 × standard deviations). Non-twitching isolates (area (cm^2^)≤ ODc), weak twitching [ODc < area (cm^2^) ≤ 2 × ODc], moderate twitching [2 × ODc < area (cm^2^)≤ 4 × ODc], and strong twitching (OD > 4 × ODc).

### Statistical analysis

Student’s t-test was performed to determine the statistical significance between clinical and environmental bacteria groups by analysing *S. maltophilia* isolates biofilm formation and twitching motility.

For combined virulence trait evaluation, Principal Component Analysis was performed using XLSTAT Statistical software, data on *S. maltophilia* growth rate, biofilm formation, and twitching motility at 37°C were used for the analysis.

## Results

### Collection of *S. maltophilia* isolates

Clinical *S. maltophilia* isolates were collected from infected patients in several tertiary care centres in Lithuania between 2017 and 2021 (Supplementary Table S1). Specific information concerning the patient’s infection site and outcome was not disclosed at the time of collection and was not available. A clinical *S. maltophilia* reference strain, D457 [12] was also included in this study. In total, 34 clinical *S. maltophilia* isolates were analysed.

Soil samples from various parts of Lithuania were collected to obtain a diverse collection of environmental *S. maltophilia* isolates (Figure 1(a)). VIA medium with mannitol-bromothymol blue indicator system was used for selective *S. maltophilia* isolation (Figure 1(b)).

In total, 38 soil samples were collected in this study, of which 23 samples resulted in the growth of mannitol fermentation-positive colonies of various morphologies (Figure 1(b)). Fifteen soil samples did not contain bacteria able to grow on selective VIA medium; of these, 11 samples were collected from soil with high sand content or otherwise low organic matter content. Out of total 190 isolated mannitol-fermentation positive colonies, 138 belonged to genus *Stenotrophomonas*, as confirmed by positive PCR analysis using primers SM1-SM4. Of these 138 isolates, 39 had unique genotyping profiles identified by RAPD-PCR. Sequencing of the 16S rRNA gene revealed 23 unique isolates belonging to *S. maltophilia* (Figure 1(c)). Previously isolated environmental isolates were also added to the study (16 isolates identified as *S. maltophilia* from soil and fish (Supplementary Table S1)) as well as a reference environmental strain R551–3. In total, 40 environmental *S. maltophilia* isolates were analysed in this study [12].

Interestingly, 23s rRNA specific primers SM1 and SM4 are described as able to select only the species *S. maltophilia* [21]. However, by analysing our environmental samples, we identified that SM1-SM4 primers also target *S. rhizophila* and *Stenotrophomonas bentatonica*, indicating that these primers should be considered genus (not species) specific. This is likely because *Stenotrophomonas* present in clinical settings is represented by only one species, *S. maltophilia*.

### Genetic relatedness of *S. maltophilia* isolates

**Figure 2.**
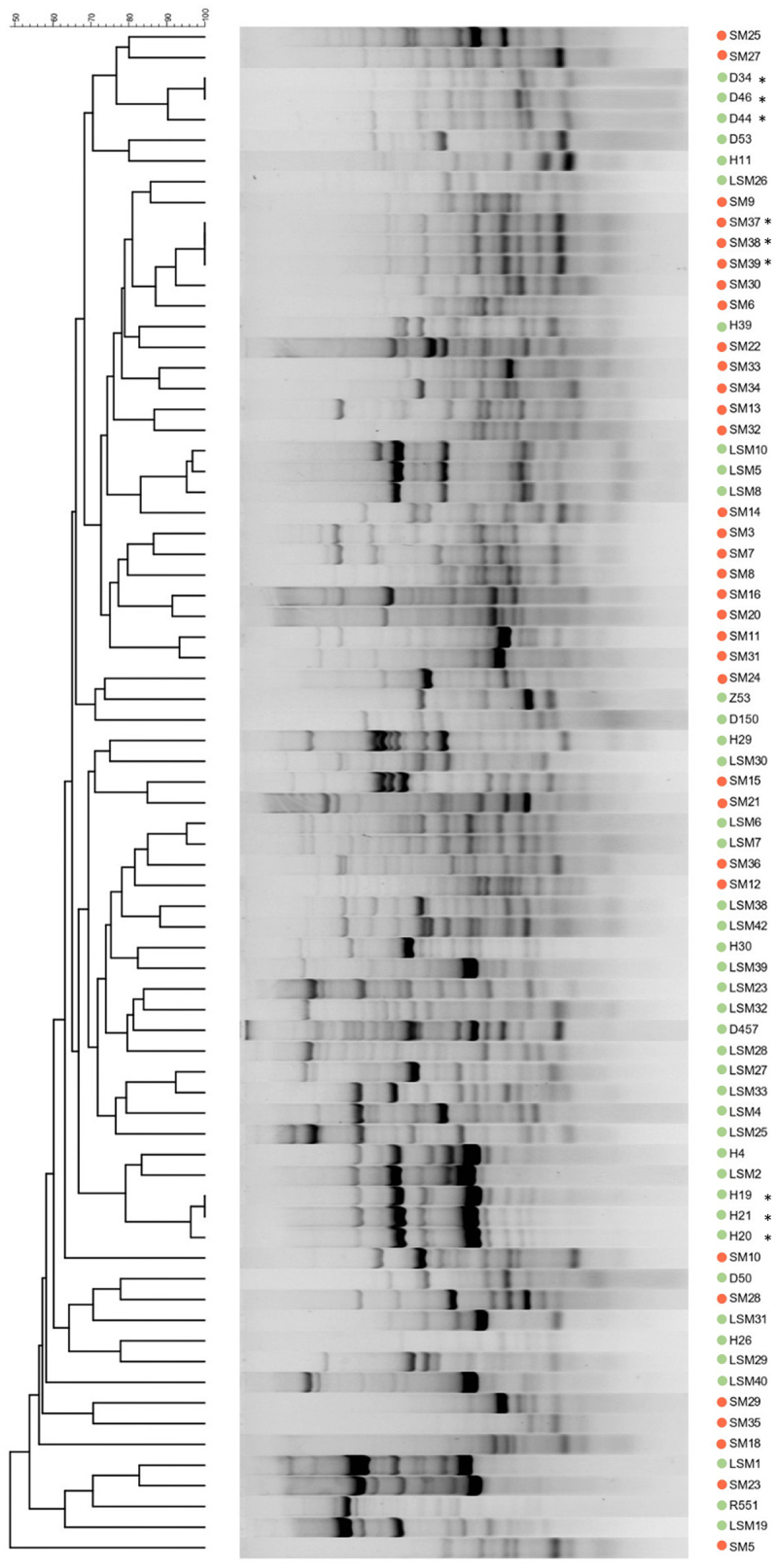
Genetic relatedness of clinical and environmental *S. maltophilia* isolates. BOX-PCR profiles were compared using GelCompar II software (Applied Maths) with Dice coefficient of 1%, band tolerance 1.5% using the UPGMA method, dendrogram is shown on the left. Red and green circles represent clinical and environmental *S. maltophilia* isolates, respectively. * indicates identical genotypic profiles between marked neighbouring isolates.

Genetic relatedness between the clinical and environmental *S. maltophilia* isolates was assessed by genotyping using BOX-PCR followed by electrophoresis in 2% agarose gel and band pattern analysis using GelComparII software. The results showed broad genotypic variation among the analysed bacteria (Figure 2). The genotyping patterns highly varied within the clinical and environmental isolate groups, and representatives of both did not fall into distinct clusters. However, the dendrogram indicated that several isolates grouped into high similarity clusters (isolates SM37, SM38, and SM39 (collected at the same hospital) had identical profiles; environment isolates D34, D44, D46 and H19, H20, H21 showed highly similar profiles) (Figure 2). Notably, the majority of clinical isolates presented distinct genotypic profiles, indicating that the isolates causing infections in different patients were not clones of one or several pathogenic clinical strains.

### The prevalence of virulence-related genes

To determine the genetically encoded virulence factors of *S. maltophilia* isolates, we investigated the prevalence of genes associated with adhesion (*afaD, papD*), biofilm formation, motility (*spgM, rmlA, fliA, smf1, rpfF, ax21, fliC, pilU*), and extracellular enzyme synthesis (*stmpr1, stmpr2, lip, smlt3773, plsN1*) (Table 1, Supplementary Fig S1). Genetic determinants encoding products associated with secretion systems, toxins, and iron acquisition were also assessed. These included the conservative genes *gspD, virB, tpsB* and *hcp* of type I, IV, V, and VI *S. maltophilia* secretion systems, respectively. *S. maltophilia* haemolysin [8] gene *hly* and Zonula occludens toxin [27] gene *zot* were also analysed. Iron was previously found to play an important role in *S. maltophilia* virulence regulation [28,29], therefore, genes related to iron acquisition (*feSR, hemO/HO, hyp1, hmuT* and *fur*) were also included in the study.

**Table 1.** Virulence-related genes detected in *S. maltophilia* isolates.

Function	Virulence genes	Prevalence of virulence related genes (%)	
		Environmental (n = 40)	Clinical (n = 34)
Biofilm and motility	*spgM*	80	91.2
	*rmlA*	95	100
	*fliA*	90	79.4
	*smf1*	40	100
	*rpfF*	5	73.5
	*ax21*	95	94.1
	*fliC*	100	100
	*pilU*	100	97.1
Toxins	*hly*	52.5	97.1
	*zot*	0	23.5
Adhesion	*afaD*	0	55.9
	*papD*	30	88.2
Secretion systems	*hcp*	10	11.8
	*gspD*	85	82.4
	*virB*	60	79.4
	*tpsB*	2.5	26.5
Extracellular enzymes	*stmpr1*	2.5	73.5
	*stmpr2*	97.5	97.1
	*lip*	20	67.6
	*smlt3773*	97.5	100
	*plsN1*	45	88.2
Iron acquisition	*feSR*	95	100
	*hemO/HO*	67.5	85.3
	*hyp1*	72.5	97.1
	*hmuT*	10	79.4
	*fur*	100	100

Of the 26 genes analysed, 15 (*spgM, rmlA, fliA, ax21, fliC, pilU, hcp, gspD, virB, stmpr2, smlt3773, feSR, hemO/HO, hyp1, fur*) were relatively similar in both the environmental and clinical isolates. However, some of the analysed genes (*smf1, rpfF, hly, zot, afaD, papD, tpsB, stmpr1, lip, plsN1, hmuT*) were significantly more prevalent in clinical isolates (Table 2, Supplementary Fig S1). On average, 21 out of 26 virulence-related genes were found to be present in clinical isolates, whereas only 15 were observed in environmental isolates (Supplementary Fig S1).

**Table 2.** Antibiotic resistance and integrase genes detected in *S. maltophilia* isolates.

Antibiotic class	Resistance genes	Prevalence of antibiotic resistance genes (%)	
		Environmental (n = 40)	Clinical (n = 34)
β-lactams	*bla*_L1_	37.5	97.1
	*bla*_L2_	92.5	94.1
	*bla*_shv_	0	0
	*bla*_IMP_	0	0
	*bla*_VIM_	0	0
Aminoglycosides	*aph(9)*	95	100
	*aph(3)*	80	70.6
	*aph(6)*	87.5	100
	*ant(2’’)Ia*	0	5.9
	*aac(6’)-Ib*	0	0
	*aac(3)IV*	0	0
	*clpA*	0	76.5
	*armA*	0	0
Sulfonamides	*sul1*	0	5.9
	*sul2*	0	0
Quinolones	*floR*	25	0
	*smqnr*	5	64.7
Trimethoprim	*dfrA1*	0	0
	*dfrA17*	0	0
	*dfrA5*	0	0
Mobile elements	*int1*	0	8.8
	*int2*	0	0

### The prevalence of antibiotic resistance genes and integrons

*S. maltophilia* possesses numerous antibiotic resistance genes [7]. In this study, we selected the most commonly analysed *S. maltophilia* genes conferring resistance to β-lactams (*bla*_L1_, *bla*_L2_), aminoglycosides (*aph(9), aph(3)* and *aph(6))*, sulphonamides (*sul1* and *sul2)*, quinolones (*smqnr*), and trimethoprim class antibiotics (*dfrA1, dfrA17* and *dfrA5*). Moreover, antibiotic resistance genes reported to be prevalent in the environment or animals (*floR* gene responsible for resistance to florfenicol) [30], genes indirectly responsible for resistance to aminoglycoside class antibiotics (*armA* and *clpA*) [31,32], and β-lactam and aminoglycoside resistance genes reported to spread across different bacterial species (*bla*_shv_, *bla*_VIM_, *bla*_IMP_, *ant(2’)Ia, aac(6”)-Ib and aac(3)IV*) were also included in the study. Gene mobility through integron structures is one of the most commonly reported mechanisms of antibiotic resistance gene spread in *S. maltophilia* [33] therefore, class I and class II integrase genes (*int1* and *int2*) were also analysed.

*bla*_L1_ gene was found in 97.1% of clinical *S. maltophilia* isolates and only in 37.5% environmental isolates, while prevalence of *bla*_L2_ gene was similar in the bacteria of both origins (Table 2, Supplementary Fig S2). The aminoglycoside resistance genes *aph(9), aph(3)* and *aph(6)* were also highly abundant in both groups. However, aminoglycoside resistance-related proteases encoding *clpA* gene were exclusively found in clinical *S. maltophilia* isolates. Only two clinical isolates (5.9%) harboured trimethoprim-sulfamethoxazole resistance gene *sul1*. *sul2*, *dfrA1, dfrA17* and *dfrA5* genes were not detected in our collection. The quinolone resistance gene *smqnr* was significantly more abundant in the clinical isolates, whereas *floR* gene was present only in the environmental strains (Table 2, Supplementary Fig S2).

Analysis of class I and II integrase genes in *S. maltophilia* revealed that only three clinical isolates encoded class I integrases. Integron gene cassette analysis of integrase-positive isolates found that two isolates had integrons harbouring aminoglycoside nucleotidyltransferase gene *ant(2’’)Ia* as determined by Sanger sequencing, while the third isolate carried an integron without any inserted gene cassette.

Although we identified some differences in the prevalence of virulence-related genes between the analysed clinical and environmental isolates of *S. maltophilia* (most notably in virulence-related genes *smf1, rpfF*, *afaD, stmpr1, hmuT* and antibiotic resistance genes *bla*_L1_,*clpA, smqnr*), the majority of the analysed genes were detected in both clinical and environmental isolates. As we were unable to separate the pathogenic and environmental isolates using a genotypic approach, we proceeded with the analysis of phenotypic virulence-related traits.

### Antibiotic resistance of *S. maltophilia* isolates

**Figure 3.**
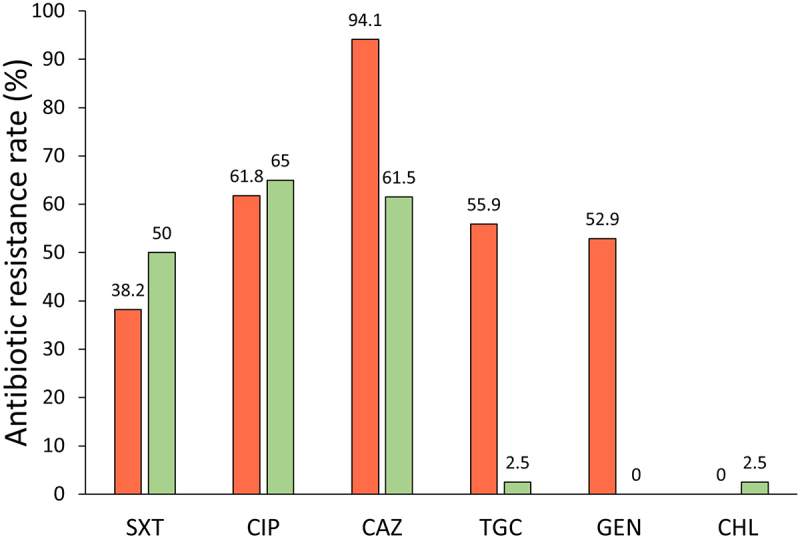
Phenotypic resistance of environmental and clinical *S. maltophilia* isolates to most commonly used antibiotics. Susceptibility assays were performed by standard disc diffusion method, results were interpreted using CLSI 2020 breakpoints. Isolates with resistant and intermediate resistant phenotypes were grouped as resistant. Resistance data of clinical and environmental *S. maltophilia* isolates are shown in red and green bars, respectively. SXT – trimethoprim-sulfamethoxazole, CIP – ciprofloxacin, CAZ – ceftazidime, TGC – tigecycline, GEN – gentamicin, CHL – chloramphenicol.

The expression of antibiotic resistance genes is regulated by a variety of factors, and despite the bacteria possessing the antibiotic resistance gene, it may not be expressed and may not exhibit the phenotype. In contrast, even if a known antibiotic resistance gene was not found in the analysed isolates, other resistance mechanisms (e.g. efflux pumps) could influence phenotypic resistance. Therefore, it is important to analyse both known genetic antibiotic resistance determinants and phenotypic resistance of the isolates. To our knowledge, there is no information on the antibiotic resistance of *S. maltophilia* isolates collected in Lithuanian hospitals or the natural environment; therefore, we analysed the resistance of our collected *S. maltophilia* isolates to various antibiotics. The most commonly used antibiotics against *S. maltophilia* worldwide were selected: trimethoprim-sulfamethoxazole (SXT), ciprofloxacin (CIP), tigecycline (TGC), ceftazidime (CAZ), gentamicin (GEN), and chloramphenicol (CHL) (Figure 3, Supplementary Fig S3).

Results showed that 38.2% of the clinical and 50% of the environmental *S. maltophilia* isolates were resistant or had an intermediate resistance to SXT. CIP resistance rates were high in both clinical and environmental isolates, reaching 61.8% and 65%, respectively. Resistance to β-lactam antibiotic CAZ was extremely high in clinical isolates (94.1%) and relatively high in environmental isolates (61.5%). GEN resistant isolates were found only in the clinical isolate group showing resistance rates of 52.9%, TGC resistance rates was 55.9% in clinical isolates, and only 2.5% in environmental bacteria. Interestingly, 100% of clinical isolates and 97.5% of environmental isolates were susceptible to CHL (Figure 3, Supplementary Fig S3).

Majority of environmental isolates were resistant to one or two antibiotics (52.5%), while 27.5% were multidrug resistant (resistant to three or more antibiotic classes), and 20% were sensitive to all antimicrobials tested. The majority of clinical isolates were multidrug resistant (58.8%), while the remaining isolates (41.2%) were resistant to one or two antibiotics (Supplementary Fig S3).

Altogether, both clinical and environmental *S. maltophilia* isolates were highly resistant to SXT, CIP, and CAZ, and sensitive to CHL. However, resistance to TGC and GEN was more characteristic of the clinical *S. maltophilia* isolates.

### The growth rate of *S. maltophilia* isolates

**Figure 4.**
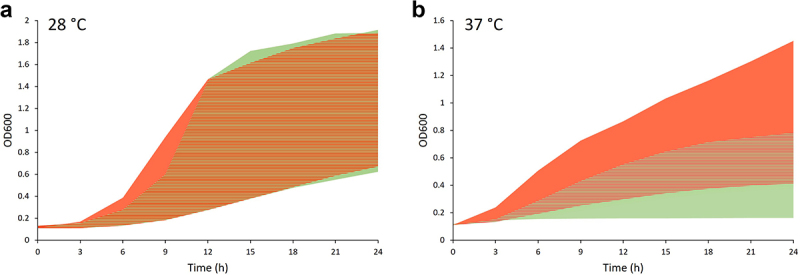
The growth rates of *S. maltophilia* isolates at 28°C (a) and 37°C (b) temperatures. The coloured areas represent combined growth curves of all analysed individual isolates. Red and green areas indicate the area of growth curves of clinical and environmental isolates, respectively. Nutrient rich TSB medium was used for cultivation.

The ability of pathogens to survive and grow at the host body temperature is considered the first and most important trait that enables bacteria to potentially cause infection [18]. In order to assess the growth potential of the analysed *S. maltophilia* at host body temperature and compare it with the ability to grow in the environment, we aimed to compare the growth rates of the isolates at 28°C and 37°C temperatures (Figure 4, Supplementary Fig S4.).

At 28°C, isolates of clinical and environmental origin displayed similar variation in growth profiles, as evident from the nearly total overlap of the growth curves of individual isolates of both groups (Figure 4(a)). However, when grown at 37°C, a clear difference was observed between the growth rates of clinical and environmental isolates (Figure 4(b)). Although the isolates of both origins were able to multiply at 37°C, the clinical isolates displayed a more prominent growth rate compared to the environmental isolates, as can be judged from the increase in OD_600_ over a period of 24 h (Figure 4(b)).

### The biofilm formation ability of *S. maltophilia* isolates

**Figure 5.**
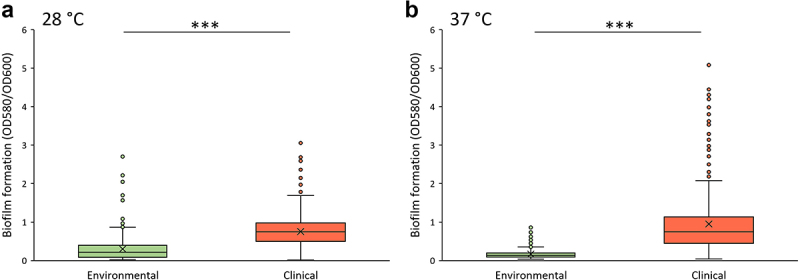
The biofilm formation ability of *S. maltophilia* isolates at 28°C (a) and 37°C (b) temperatures. The biofilm formed was measured using crystal violet dye assay. Red and green colours indicate the biofilm formation of clinical and environmental isolates, respectively. Boxes indicate upper and lower quartiles, whiskers indicate minimum and maximum values excluding outliers, circles mark outliers, and crosses indicate mean values. *** indicate statistical significance (*p* < 0.005) between the groups, evaluated by t-test.

As we observed significant difference between the growth rates of clinical and environmental *S. maltophilia* isolates at 37°C, we further proceeded with the comparison of virulence associated phenotypic traits (biofilm formation and twitching motility) at environmental and host body temperatures. Biofilm formation has been reported to be one of the major virulence traits of *S. maltophilia* [9]. *S.* *maltophilia* can form biofilms on abiotic surfaces such as glass or plastic (e.g. intravenous catheters, respiratory tubes, prosthetic devices) as well as on biotic surfaces, such as lung cells, tracheal cells, or host tissues [34]. The ability to form biofilms on intravenous devices or host tissues may be influenced by the host body temperature. Therefore, next we analysed the ability of clinical and environmental *S. maltophilia* isolates to form biofilms at 28°C and 37°C (Figure 5, Supplementary Figure S5).

Our results showed a significant difference in the ability to form biofilms between clinical and environmental isolates at both 28°C and 37°C temperatures (Figure 5, Supplementary Fig S5). The majority of environmental isolates were weak biofilm producers at 28°C temperature, whereas at 37°C the majority of isolates did not form biofilms (Table 3). In contrast, 62% and 53% of the clinical isolates produced strong biofilm at 28°C and 37°C temperatures, respectively. Only two clinical isolates (6%) were unable to form biofilms at either temperature (Table 3).

**Table 3.** Biofilm formation ability overview of clinical and environmental *S. maltophilia* isolates.

*S. maltophilia* isolates	Temperature	Non-biofilm producer	Weak-producer	Moderate-producer	Strong producer
Environmental	28°C	14 (35%)	15 (37.5%)	9 (22.9%)	2 (5%)
	37°C	28 (70%)	10 (25%)	2 (5%)	0 (0%)
Clinical	28°C	2 (6%)	1 (3%)	10 (29%)	21 (62%)
	37°C	2 (6%)	3 (9%)	11 (32%)	18 (53%)

### Twitching motility of *S. maltophilia* isolates

**Figure 6.**
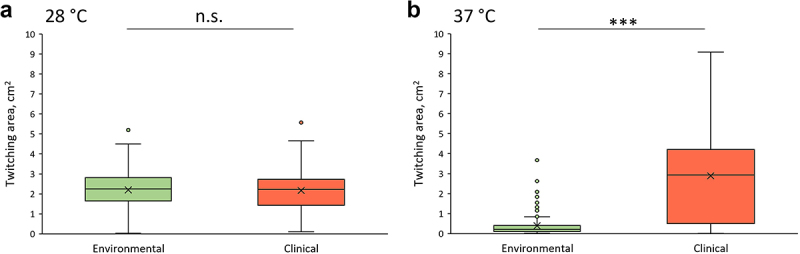
The twitching motility of *S. maltophilia* isolates at 28°C (a) and 37°C (b) temperatures. Evaluation was performed using semi-solid medium in a humid environment. Red and green colour indicate the twitching motility of clinical and environmental isolates, respectively. Twitching zones were measured in cm^2^. Boxes indicate upper and lower quartiles, whiskers indicate minimum and maximum values excluding outliers, circles mark outliers, crosses indicate mean values. *** indicate statistical significance (*p* < 0.005) between the groups, evaluated by t-test, n.s. indicates no statistical significance between the groups.

Bacterial motility plays an important role in the process of infection, allowing pathogens to spread across biotic and abiotic surfaces, migrate to favourable environments, and colonise the host [35,36]. It has been reported that *S. maltophilia* isolates use twitching motility; however, the twitching ability varies greatly depending on the isolate [37,38]. Only a few studies have analysed the twitching motility of the environmental *S. maltophilia* isolates [11,37], therefore to gain insight into phenotypic virulence-related features of *S. maltophilia* isolates, we analysed their twitching motility at 28°C and 37°C (Figure 6, Supplementary Figure S6).

Our data revealed that the majority of clinical and environmental isolates were able to display twitching motility at 28°C, and no significant differences were observed between the groups (Figure 6, Supplementary Figure S6). However, a clear difference was evident at 37°C, as the majority of the environmental isolates lost their strong-twitching ability, whereas clinical isolates retained and even increased this capacity (Table 4, Figure 6, Supplementary Figure S6).

**Table 4.** Twitching motility ability overview of clinical and environmental *S. maltophilia* isolates.

*S. maltophilia* isolates	Temperature	Non-twitching	Weak-twitching	Moderate-twitching	Strong twitching
Environmental	28°C	1 (2.5%)	0 (0%)	2 (5%)	37 (92.5%)
	37°C	15 (37.5%)	14 (35%)	7 (17.5%)	4 (10%)
Clinical	28°C	1 (3%)	1 (3%)	1 (3%)	31 (91%)
	37°C	1 (3%)	5 (15%)	3 (9%)	25 (73%)

### Combined virulence trait evaluation

**Figure 7.**
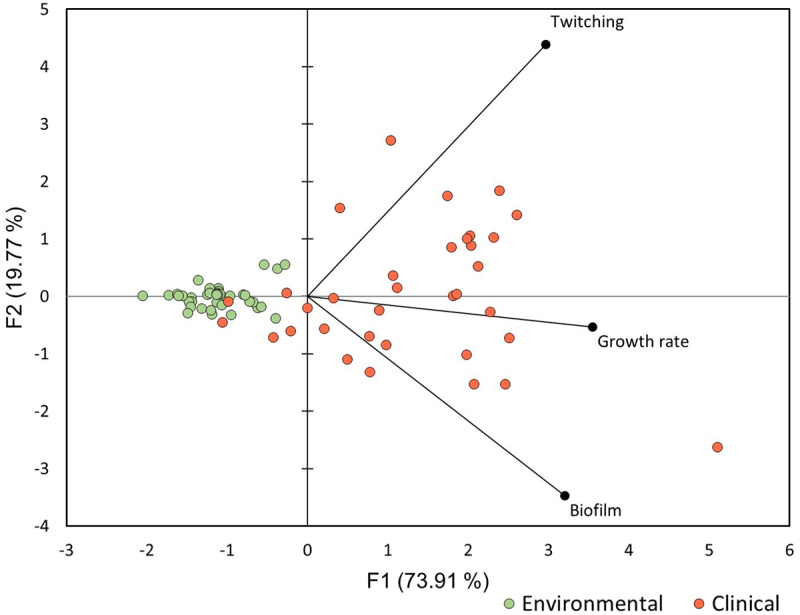
The principal component analysis of virulence related traits (growth rate, biofilm formation and twitching motility at 37°C) of clinical and environmental isolates of *S. maltophilia.*

This study revealed significant differences in the ability of *S. maltophilia* to grow and express virulence-related features (biofilm formation and twitching motility) at human body temperature (Figures 4(b), 5(b) and 6(b)). In order to perform combined evaluation of these features for clinical and environmental isolates, we performed Principal Component Analysis using data on biofilm formation, twitching motility, and growth rate at 37°C (Figure 7).

The results showed that environmental isolates of *S. maltophilia* formed a concentrated cluster, whereas clinical isolates were significantly more distributed against the F1 and F2 axes (Figure 7). F1 and F2 axes combined 93.68% of variation in the analysed *S. maltophilia* isolates. Only a few clinical isolates overlapped with the cluster of the environmental *S. maltophilia*, indicating that these clinical isolates displayed similar virulence traits at 37°C as the environmental isolates.

## Discussion

*S. maltophilia* is considered a newly emerging pathogen of concern, as in recent years the number of infections caused by this bacterium has increased significantly [6,39]. *S. maltophilia* is highly abundant in various natural sources [1], therefore, it is important to understand how many environmental *S. maltophilia* variants could potentially cause infections. In this study we aimed to determine the major traits and conditions distinguishing clinical and environmental *S. maltophilia* isolates.

Characterization of environmental samples collected for this study revealed that bacteria belonging to the species *S. maltophilia* were found in 60% of the obtained soil samples showing that this bacterium is highly abundant in soil. Majority of the clinical and environmental isolates showed highly distant genetic profiles, indicating a lack of clonal spread. High genomic variation in *S. maltophilia* isolates has been reported previously in other studies [40–43], and is one of the major factors obstructing the unambiguous differentiation of pathogenic and harmless *S. maltophilia* variants [16].

### Antibiotic resistance

Our study showed that resistance to SXT, the first line antibiotic against *S. maltophilia*, was 38% among clinical isolates, which is similar to SXT resistance rates reported by other studies [9,44]. Resistance of environmental isolates to SXT was also high (50%); however, similar results were observed in other studies analysing *S. maltophilia* isolates from soil [45]. Interestingly, despite the relatively high SXT resistance rates, the most common genes conferring resistance to sulphonamides were not found in the analysed *S. maltophilia* isolates. We also did not find any integron cassettes with *sul* or *dfr* genes responsible for resistance to sulphonamide class antibiotics, which are typically present in *S. maltophilia* integron structures [46]. This suggests that other resistance mechanisms, such as antibiotic elimination through efflux pumps may be responsible for resistance to sulphonamide-class antibiotics in the isolates investigated, as it was showed to contribute to *S. maltophilia* resistance to SXT before [47,48]. A significant difference in resistance to GEN and TGC was observed between clinical and environmental *S. maltophilia*. The sensitivity to GEN of environmental *S. maltophilia* isolates might be related to the increase in the temperature to 37°C affecting the cell wall structure of *S. maltophilia*, as it has been shown that host body temperature alter the lipopolysaccharide composition of *S. maltophilia* resulting in altered sensitivity to aminoglycoside class antibiotics [49]. The high TGC resistance rate among clinical *S. maltophilia* could indicate TGC pressure in clinical settings and might be determined by various antibiotic resistance mechanisms, especially *smeDEF* efflux pump [50]. Notably, CHL was effective against all analysed clinical and environmental isolates, and only the reference strain R551–3 displayed intermediate resistance to CHL. This shows the great potential of using CHL against otherwise resistant *S. maltophilia*, even though this antibiotic is not currently used for the treatment of infections due to its side effects [51].

### Antibiotic resistance genes

Both aminoglycoside and β-lactam resistance genes are highly prevalent in *S. maltophilia* isolates [33], and our results showed similar trends among the isolates collected in Lithuania. Interestingly, the quinolone resistance gene *smqnr* was detected only in the clinical isolates, whereas *floR* gene was found in the environmental bacteria only. *smqnr* gene is reported to be found in clinical *S. maltophilia* isolates at similar rates in other studies (study from Iraq showed that *smqnr* gene was found in 65% of clinical isolates [52], study from Mexico showed that *smqnr* gene was one of the most abundant genes and was found in 83% of analysed isolates) [37]. Florfenicol can be used in Lithuanian agriculture [53], and antibiotic pressure in the environment could be responsible for selection of *floR* in environmental *S. maltophilia* isolates. The protease encoding *clpA* gene was exclusively found in clinical *S. maltophilia* isolates. Although the mechanisms by which *clpA* protease contributes to antibiotic resistance are still unknown, *clpA* has been demonstrated to be responsible for resistance to aminoglycoside class antibiotics associated with *smeYZ* efflux pump [32].

### Virulence-related genes

Biofilm and motility-associated genes *spgM, rmlA, fliA, ax21, fliC, pilU* were highly prevalent in all isolates; however, *smf1* and *rpfF* genes were significantly more abundant in the group of clinical origin. *smf1* and *rpfF* genes are directly related to the ability of *S. maltophilia* to form biofilms [54,55]. Our analysis of *smf1* and *rpfF* gene distribution and biofilm formation also confirmed this association, as *smf1* and *rpfF* genes and the ability to form biofilms were more prevalent in clinical isolates. Adhesion is one of the first steps in biofilm formation, and *S. maltophilia afaD* and *papD* genes, coding for adhesin and pilus assembly protein, respectively, are associated with biofilm formation [52]. In our study, *afaD* and *papD* were more prevalent in clinical *S. maltophilia* which could be related to the more pronounced capacity of biofilm formation among clinical *S. maltophilia*. However, in order to evaluate gene functional involvement in the phenotype, additional studies of gene expression would be necessary.

Extracellular enzymes are known to play major roles in *S. maltophilia* virulence, helping to degrade various substances, including human host components [9,56]. Stmpr1 protease, which in our study was highly abundant only in clinical isolates, has been shown to degrade collagen, fibronectin, α_1_-antitrypsin, α_2_-macroglobulin, and polyclonal IgG from human serum [56], indicating its pathogenic significance in clinical *S. maltophilia*. Iron acquisition related genes were highly abundant in both clinical and environmental isolates with exception of *hmuT* gene, which was found only in 10% of environmental isolates. Free iron is a limited resource, especially in the host body, and a high prevalence of iron acquisition related genes shows the importance of iron acquisition in both the natural environment and clinical niches. Surprisingly, *zot* toxin gene was found in 24% of clinical *S. maltophilia* isolates, contrary to the previous studies where *zot* gene was not detected [37,57]. Although the function of *S. maltophilia* zot toxin (homologue of *Vibrio cholerae* toxin impairing intercellular junctions in host cells [58] is not known, it was previously reported to be found in *S. maltophilia* bacteriophage genome [27].

### Growth and virulence-related phenotypes in host body temperature

A crucial stress factor that pathogens of the environmental origin have to adapt to in order to cause infections in humans is a change from the environmental temperature range (typically 22–30°C) to the temperature of human host body (37°C) [18]. Because survival and multiplication in the host organism are required to cause infection [59], all pathogenic bacteria capable of causing human infections should be able to grow at 37°C. We have shown that the average growth rate of clinical *S. maltophilia* at 37°C was significantly higher that of environmental isolates, indicating that clinical isolates are well adapted to grow at the host body temperature. Nearly one third (28%) of the environmental isolates exhibited lower growth rate under these conditions than the slowest growing clinical *S. maltophilia* strain.

Clinical and environmental *S. maltophilia* isolates are known to produce biofilms, although biofilm formation capacity of the environmental isolates is typically studied in the temperature range of 25–30°C [60,61]. In this study, we demonstrated that more than a half of clinical *S. maltophilia* isolates were strong biofilm producers at both 28°C and 37°C. However, 70% of environmental bacteria were unable to form biofilms at 37°C and were categorized as non-biofilm producers. These results imply that the biofilm formation ability of environmental *S. maltophilia* isolates is highly affected by host body temperature, while the clinical isolates are more adapted to it. The motility of *S. maltophilia* is known to be affected by body temperature [18]. Patil et al. have found that genes responsible for bacterial motility as well as motility phenotype were downregulated at 37°C temperature comparing to the transcriptome data of bacteria grown at 28°C. However, this analysis is limited to a single clinical isolate of *S. maltophilia*. In this study, we showed that the majority of environmental *S. maltophilia* isolates lost their strong twitching ability at the host body temperature, while the majority of the clinical isolates retained this feature. Moreover, more than half of the clinical isolates increased their twitching area at 37°C temperature compared to that displayed at 28°C, indicating that twitching motility may be present when isolates are exposed to temperature of the human body.

Combined evaluation of growth- and virulence-related phenotypes at 37°C revealed that clinical and environmental *S. maltophilia* isolates formed separate clusters, showing that temperature tolerance was more characteristic of clinical *S. maltophilia* and virulence related traits were more pronounced. However, we identified that some clinical isolates overlapped with the cluster of environmental *S. maltophilia*, indicating that these isolates displayed traits more similar to the environmental bacteria and might have recently originated from an environmental origin. Our results and those of other authors suggest that temperature tolerance could be a major factor distinguishing pathogenic and non-pathogenic *S. maltophilia* isolates [19]. Therefore, similarities in temperature tolerance between clinical and environmental isolates alarms of a potential reservoirs of human pathogens in the natural environment. It is widely acknowledged that climate change can create an environment in which microorganisms would adapt to survive and thrive at higher temperatures, thereby enabling harmless bacteria to adapt to the temperature of the human body. The rhizosphere is a known reservoir of potentially pathogenic bacteria with various virulence features [62], and the lack of temperature tolerance is the limiting factor for them to cause infections in humans. Therefore, natural adaptation to higher temperatures due to the warming climate might create an outburst of human infections by naturally prevalent bacteria, such as *S. maltophilia*.

## Conclusions

- Clinical and environmental *S. maltophilia* isolates display high genotypic variation. Although some differences between clinical and environmental isolates were found, most of the analysed genes were present in the isolates of both groups, suggesting that the profiles of genetic determinants are not associated with the origin of *S. maltophilia*.
- Most importantly, the bacterial growth and virulence-related phenotypes at human host body temperature were characteristic to the clinical *S. maltophilia* isolates, thus this trait could be considered a key adaptation transforming harmless *S. maltophilia* from the environment into a potential pathogen.

## Supplementary Material

Supplementary Fig 1_6.docx

Supplementary Tables 1_2.docx

## Data Availability

The data that support the findings of this study is deposited in Zenodo and can be accessed with the link: DOI 10.5281/zenodo.14007645.
