# Supplementary material for: Stenotrophomonas maltophilia of clinical origin display higher temperature tolerance comparing with environmental isolates
Source: Virulence. 2025 May 2;16(1):2498669. doi: 10.1080/21505594.2025.2498669 (PMC12064055; doi:10.1080/21505594.2025.2498669)

**Supplementary Fig 1**. Prevalence of virulence related genes in environmental and clinical isolates of *Stenotrophomonas maltophilia* collected in Lithuania. Red colour indicates clinical *S. maltophilia* isolates, green colour – environmental *S. maltophilia* isolates. Gene detection analysis was performed using standard Polymerase Chain Reaction. Dark grey square – gene detected, white square – gene not detected.

**Supplementary Fig 2.** Prevalence of antibiotic resistance genes in environmental and clinical isolates of *Stenotrophomonas maltophilia* collected in Lithuania. Red colour indicates clinical *S. maltophilia* isolates, green colour – environmental *S. maltophilia* isolates. Gene detection analysis was performed using standard Polymerase Chain Reaction. Dark grey square – gene detected, white square – gene not detected.

**Supplementary Fig 3.** Clinical and environmental *S. maltophilia* isolates antibiotic resistance phenotype. Resistance evaluation was performed using standard disc diffusion method, interpretation done using Clinical and Laboratory Standards Institute 2020 breakpoints. Red colour indicates clinical *S. maltophilia* isolates, green colour – environmental *S. maltophilia* isolates. Dark grey square – resistant, light grey square – intermediate resistant, white square – sensitive. SXT – trimethoprim-sulfamethoxazole, CIP – ciprofloxacin, CAZ – ceftazidime, TGC – tigecycline, GEN gentamicin, CHL – chloramphenicol. * because of limited bacteria growth disc diffusion plates were kept at 37 °C for 48h; - due to lack of growth antibiotic resistance evaluation not done.

**Supplementary Fig 4**. *S. maltophilia* ability to grow at environmental (28 °C) and host body (37 °C) temperatures. Growth evaluation was performed using nutrient rich Tryptic Soy Broth (TSB) medium, isolates growth (OD_600_) was measured in a Tecan Infinite M200 Pro plate reader at 28 °C or 37 °C for 24h (with shaking). Green line indicates environmental isolates growth, red line – clinical isolates growth. Three independent experiments were performed for each isolate growth assessment at both environmental and host body temperatures, each line represents average of one isolates growth.


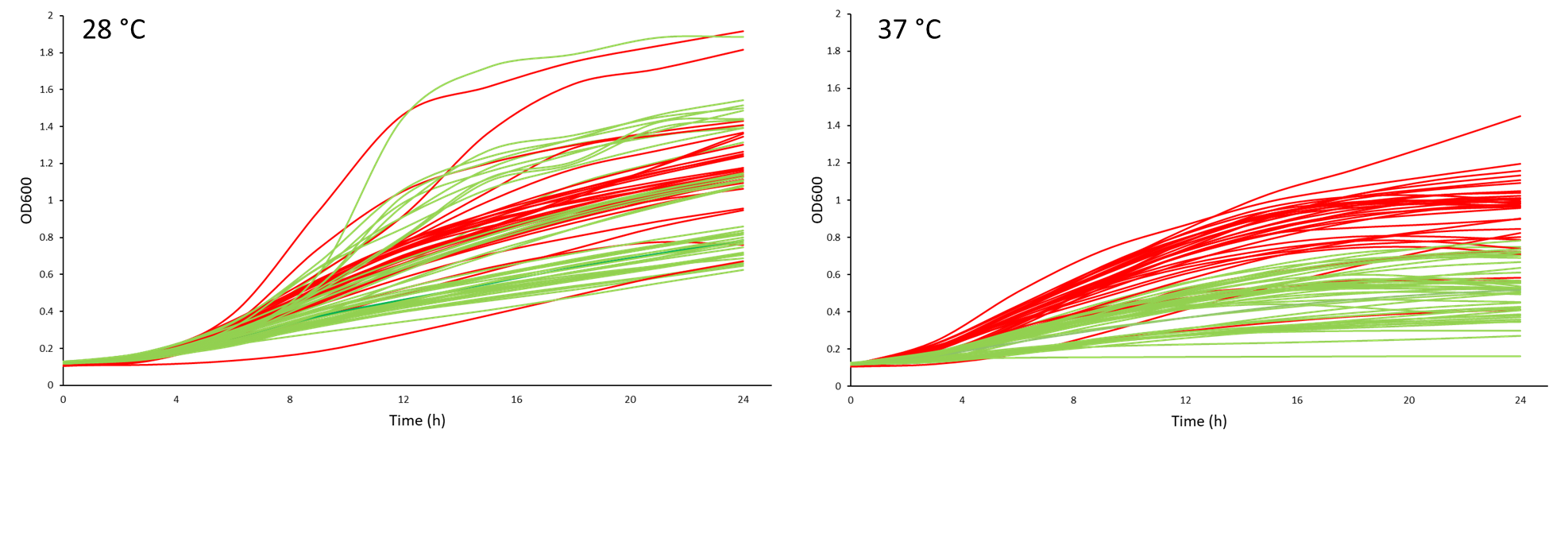


**Supplementary Fig 5**. *S. maltophilia* ability to form biofilm at environmental (28 °C) and host body (37 °C) temperatures. Red colour indicates clinical *S. maltophilia* isolates, green colour – environmental *S. maltophilia* isolates. Biofilms were grown at 28 °C and 37 °C temperatures, evaluation was performed using Crystal violet dye assay. Boxes indicate upper and lower quartiles, whiskers indicate minimum and maximum values excluding outliers, circles marks outliers and crosses indicate mean values.


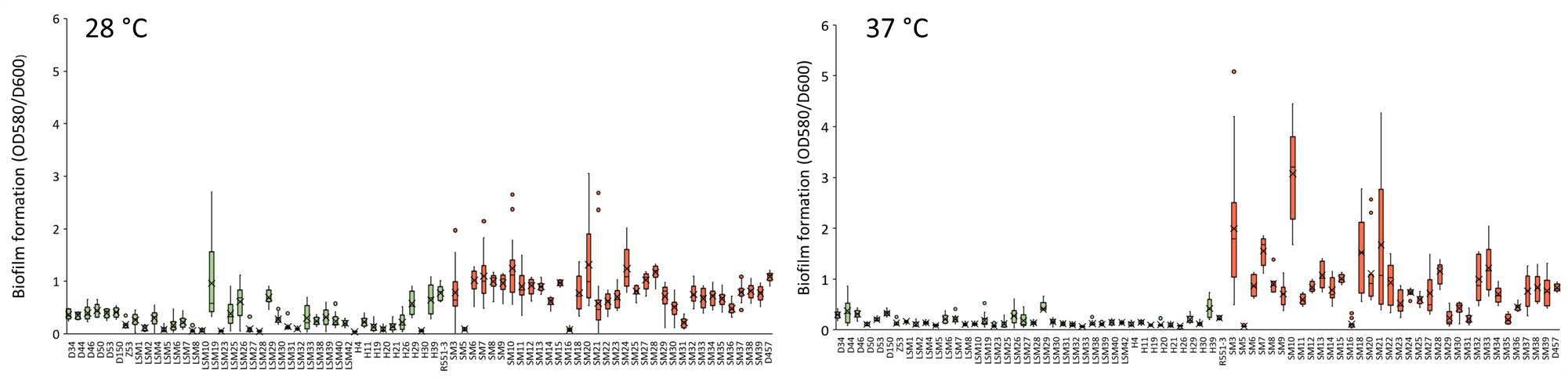


**Supplementary Fig 6**. *S. maltophilia* twitching motility at environmental (28 °C) and host body (37 °C) temperatures. Red colour indicates clinical *S. maltophilia* isolates, green colour – environmental *S. maltophilia* isolates. Twitching motility evaluation was performed using semi-solid medium in a humid environment at 28 °C and 37 °C temperatures, twitching zones were measured in cm^2^. Boxes indicate upper and lower quartiles, whiskers indicate minimum and maximum values excluding outliers, circles marks outliers and crosses indicate mean values.


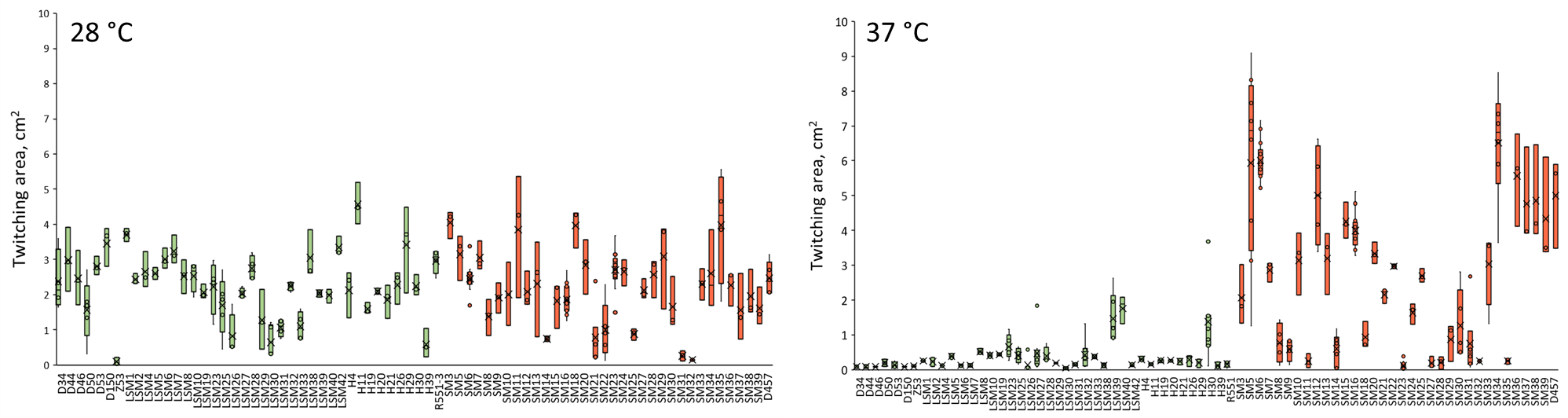

Supplement: Supplementary Fig 1_6.docx [file KVIR_A_2498669_SM6432.docx]
