# Supplementary material for: Stenotrophomonas maltophilia of clinical origin display higher temperature tolerance comparing with environmental isolates
Source: Virulence. 2025 May 2;16(1):2498669. doi: 10.1080/21505594.2025.2498669 (PMC12064055; doi:10.1080/21505594.2025.2498669)
Supplement: Supplementary Tables 1_2.docx [file KVIR_A_2498669_SM6431.docx]

**Supplementary Table 1.** Clinical and environmental *S. maltophilia* isolates used in this study.

| ***Stenotrophomonas maltophilia* isolate** | **Origin** | | **Source*** | | **Year of isolation** | | **Reference** | |  |
| --- | --- | --- | --- | --- | --- | --- | --- | --- | --- |
| D34 | Environmental | | Soil | | 2016 | | (Klimkaitė et al., 2020) | |  |
| D44 | Environmental | | Soil | | 2016 | | (Klimkaitė et al., 2020) | |  |
| D46 | Environmental | | Soil | | 2016 | | (Klimkaitė et al., 2020) | |  |
| D50 | Environmental | | Soil | | 2016 | | (Klimkaitė et al., 2020) | |  |
| D53 | Environmental | | Soil | | 2016 | | (Klimkaitė et al., 2020) | |  |
| D150 | Environmental | | Soil | | 2016 | | (Klimkaitė et al., 2020) | |  |
| Z53 | Environmental | | Fish | | 2016 | | (Klimkaitė et al., 2020) | |  |
| H4 | Environmental | | Fish | | 2020 | | Acquired from M. Ružauskas | |  |
| H11 | Environmental | | Fish | | 2020 | | Acquired from M. Ružauskas | |  |
| H19 | Environmental | | Fish | | 2020 | | Acquired from M. Ružauskas | |  |
| H20 | Environmental | | Fish | | 2020 | | Acquired from M. Ružauskas | |  |
| H21 | Environmental | | Fish | | 2020 | | Acquired from M. Ružauskas | |  |
| H26 | Environmental | | Fish | | 2020 | | Acquired from M. Ružauskas | |  |
| H29 | Environmental | | Fish | | 2020 | | Acquired from M. Ružauskas | |  |
| H30 | Environmental | | Fish | | 2020 | | Acquired from M. Ružauskas | |  |
| H39 | Environmental | | Fish | | 2020 | | Acquired from M. Ružauskas | |  |
| LSM1 | Environmental | | Soil | | 2021 | | This work | |  |
| LSM2 | Environmental | | Soil | | 2021 | | This work | |  |
| LSM4 | Environmental | | Soil | | 2021 | | This work | |  |
| LSM5 | Environmental | | Soil | | 2021 | | This work | |  |
| LSM6 | Environmental | | Soil | | 2021 | | This work | |  |
| LSM7 | Environmental | | Soil | | 2021 | | This work | |  |
| LSM8 | Environmental | | Soil | | 2021 | | This work | |  |
| LSM10 | Environmental | | Soil | | 2021 | | This work | |  |
| LSM19 | Environmental | | Soil | | 2021 | | This work | |  |
| LSM23 | Environmental | | Soil | | 2021 | | This work | |  |
| LSM25 | Environmental | | Soil | | 2021 | | This work | |  |
| LSM26 | Environmental | | Soil | | 2021 | | This work | |  |
| LSM27 | Environmental | | Soil | | 2021 | | This work | |  |
| LSM28 | Environmental | | Soil | | 2021 | | This work | |  |
| LSM29 | Environmental | | Soil | | 2021 | | This work | |  |
| LSM30 | Environmental | | Soil | | 2021 | | This work | |  |
| LSM31 | Environmental | | Soil | | 2021 | | This work | |  |
| LSM32 | Environmental | | Soil | | 2021 | | This work | |  |
| LSM33 | Environmental | | Soil | | 2021 | | This work | |  |
| LSM38 | Environmental | | Soil | | 2021 | | This work | |  |
| LSM39 | Environmental | | Soil | | 2021 | | This work | |  |
| LSM40 | Environmental | | Soil | | 2021 | | This work | |  |
| LSM42 | Environmental | | Soil | | 2021 | | This work | |  |
| R551-3 | Environmental | | - | | 2008 | | (Lira et al., 2017) | |  |
| SM3 | Clinical | | NCI | | 2017 | | (Klimkaitė et al., 2020) | |  |
| SM5 | Clinical | | VCCH LM | | 2018 | | (Klimkaitė et al., 2020) | |  |
| SM6 | Clinical | | VCCH LM | | 2018 | | (Klimkaitė et al., 2020) | |  |
| SM7 | Clinical | | VCCH LM | | 2018 | | (Klimkaitė et al. 2020) | |  |
| SM8 | Clinical | | NCI | | 2019 | | (Klimkaitė et al. 2020) | |  |
| SM9 | Clinical | | NCI | | 2019 | | (Klimkaitė et al. 2020) | |  |
| SM10 | Clinical | | VCCH LM | | 2019 | | (Klimkaitė et al. 2020) | |  |
| SM11 | Clinical | | VUH SK PD | | 2019 | | (Klimkaitė et al. 2020) | |  |
| SM12 | Clinical | | VUH SK PD | | 2019 | | (Klimkaitė et al. 2020) | |  |
| SM13 | Clinical | | VUH SK PD | | 2019 | | (Klimkaitė et al. 2020) | |  |
| SM14 | Clinical | | VCCH LM | | 2019 | | (Klimkaitė et al. 2020) | |  |
| SM15 | Clinical | | KCPHC | | 2019 | | (Klimkaitė et al. 2020) | |  |
| SM16 | Clinical | | VCCH LM | | 2019 | | (Klimkaitė et al. 2020) | |  |
| SM17 | Clinical | | NCI | | 2019 | | (Klimkaitė et al. 2020) | |  |
| SM18 | Clinical | | NCI | | 2019 | | (Klimkaitė et al. 2020) | |  |
| SM20 | Clinical | | NCI | | 2019 | | (Klimkaitė et al. 2020) | |  |
| SM21 | Clinical | | NCI | | 2019 | | (Klimkaitė et al. 2020) | |  |
| SM22 | Clinical | | NCI | | 2019 | | (Klimkaitė et al. 2020) | |  |
| SM23 | Clinical | | NCI | | 2019 | | (Klimkaitė et al. 2020) | |  |
| SM24 | Clinical | | NCI | | 2019 | | (Klimkaitė et al. 2020) | |  |
| SM25 | Clinical | | NCI | | 2019 | | (Klimkaitė et al. 2020) | |  |
| SM27 | Clinical | | NCI | | 2019 | | This study | |  |
| SM28 | Clinical | | VCCH LM | | 2020 | | This study | |  |
| SM29 | Clinical | | VCCH LM | | 2021 | | This study | |  |
| SM30 | Clinical | | VCCH LM | | 2021 | | This study | |  |
| SM31 | Clinical | | VCCH LM | | 2021 | | This study | |  |
| SM32 | Clinical | | VCCH LM | | 2021 | | This study | |  |
| SM33 | Clinical | | VCCH LM | | 2021 | | This study | |  |
| SM34 | Clinical | | VCCH LM | | 2021 | | This study | |  |
| SM35 | Clinical | | VCCH LM | | 2021 | | This study | |  |
| SM36 | Clinical | | VCCH LM | | 2021 | | This study | |  |
| SM37 | Clinical | | VCCH LM | | 2021 | | This study | |  |
| SM38 | Clinical | | VCCH LM | | 2021 | | This study | |  |
| SM39 | Clinical | | VCCH LM | | 2021 | | This study | |  |
| D457 | Clinical | | - | | 2012 | | (Lira et al., 2012) | |  |
|  | |  | |  | |  | |  | |

*Environmental *S. maltophilia* were isolated from soil (samples collected in the various locations of Lithuania) and fish gut; clinical isolates were collected from infected patients from several healthcare centres in Lithuania: NCI—National Cancer Institute (Lithuania); VCCH LM—Vilnius City Clinical Hospital, Laboratory of Microbiology; VUH SK PD—Vilnius University Hospital Santaros Klinikos, Pediatrics Department, Division of Infectious Diseases; KCPHC—Kaunas City Public Health Center.

**Supplementary Table 2.** Primers used in this study.

| **Primer name** | **Primer sequence 5' – 3'** | **Primer function** | **Reference** |
| --- | --- | --- | --- |
| SM1 | CAGCCTGCGAAAAGTA | Detection of bacteria from genus *Stenotrophomonas* | (Whitby et al., 2000) |
| SM4 | TTAAGCTTGCCACGAACAG |  |  |
| OPA-02 | TGCCGAGCTG | Genotyping | (Yu et al., 1997) |
| 380-7 | GGCAAGCGGG | Genotyping | (Klimkaitė et al., 2020) |
| 27F | AGAGTTTGATYMTGGCTCAG | Bacteria species identification | (Kim et al., 2012) |
| 515R | TTACCGCGGCKGCTGGCAC |  |  |
| 1492R | TACGGYTACCTTGTTACGACTT |  |  |
| BoxA1R | CTACGGCAAGGCGACGCTGACG | Genotyping | (Versalovic et al., 1994) |
| shv_F | AGGATTGACTGCCTTTTTGCG | *bla*_shv_ gene detection | (Fang et al., 2008) |
| shv_R | ATTTGCTGATTTCGCTCGGC |  |  |
| imp_F | GGAATAGAGTGGCTTAATTCTC | *bla*_IMP_ gene detection | (Kaczmarek et al., 2006) |
| imp_R | GCGGACTTTGGCCAAGCTT |  |  |
| vim_F | GCACTTCTCGCGGAGAT | *bla*_VIM_ gene detection | This work |
| vim_R | ACTGGACCGAAGCGCACTG |  |  |
| ph_F | GACASCCTGCAYTGGMTGCG | *aph(9)*gene detection | This work |
| ph_R | TAVGGGAACARSGTGAACTGC |  |  |
| aph(3)_F | CSTTCSTGAARTCGGAAGTGATCG | *aph(3)* gene detection | (Klimkaitė et al., 2023) |
| aph(3)_R | CCRCARTCRATGAAACCRCTGAA |  |  |
| aph(6)_F | NCCRCASAGRTCCGGRTTG | *aph(6)* gene detection | (Klimkaitė et al., 2023) |
| aph(6)_R | GGYGACCTGCAYCAYGACAA |  |  |
| ant(2‘‘)Ia_F | CCGCAGCTAGAATTTTG | *ant(2‘‘)Ia* gene detection | (Baseri et al., 2021) |
| ant(2‘‘)Ia_R | AGGTTGAGGTCTTGCGT |  |  |
| aac(6‘)-Ib_F | AGTACAGCATCGTGACCAACA | *aac(6‘)-Ib* gene detection | (Galimand *et* *al*., 1993) |
| aac(6‘)-Ib_R | ATGTACACGGCTGGACCATC |  |  |
| aac(3)IV_F | GATGGGCCACTTGGACTGAT | *aac(3)IV* gene detection | (Chen *et al*., 2005) |
| aac(3)IV_R | GCGCTCACAGCAGTGGTCAT |  |  |
| clpA_F | TCGGTACCGCTGCTGATCTTC | *clpA* gene detection | (Saleh *et al*., 2021) |
| clpA_R | CCGAGCTCTGCACGTGGTACA |  |  |
| armA_F | ATTCTGCCTATCCTAATTGG | *armA* gene detection | (Helmy ir Kashef, 2017) |
| armA_R | ACCTATACTTTATCGTCGTC |  |  |
| sul1_F | TCACCGAGGACTCCTTCTTC | *sul1* gene detection | (Chen et al., 2004) |
| sul1_R | CAGTCCGCCTCAGCAATATC |  |  |
| sul2_F | CCTGTTTCGTCCGACACAGA | *sul2* gene detection | (Chen et al., 2004) |
| sul2_R | GAAGCGCAGCCGCAATTCAT |  |  |
| dfrA1_F | CTTGTTAACCCTTTTGCCAGA | *dfrA1* gene detection | (Baseri et al., 2021) |
| dfrA1_R | TTGTGAAACTATCACTAATGGTAG |  |  |
| dfrA17_F | GTTAGCCTTTTTTCCAAATCTGGTATG | *dfrA17* gene detection | (Baseri et al., 2021) |
| dfrA17_R | TTGAAAATATTATTGATTTCTGCAGTG |  |  |
| dfrA5_F | ATCGTCGATATATGGAGCGTA | *dfrA5* gene detection | (Baseri et al., 2021) |
| dfrA5_R | TCCACACATACCCTGGTCCG |  |  |
| floR_F | GTTTCAGGTGGCACGAAACC | *floR* gene detection | (Lastauskienė et al., 2021) |
| floR_R | CGGACACCGTGAAGACAATA |  |  |
| qnr_F | ACACAGAACGGCTGGACTGC | *smqnr* gene detection | (Malekan et al., 2017) |
| qnr_R | TTCAACGACGTGGAGCTGTT |  |  |
| hly_F | CGTCCATTGCTTCGATCCGTG | *hly* gene detection | (Cruz-Córdova et al., 2020) |
| hly_R | GACGAAGTGGCAGACGCTG |  |  |
| zot_F | GCGTCAGTACACCGATGGTTG | *zot* gene detection | (Cruz-Córdova et al., 2020) |
| zot_R | GCAGGCAGTGTCCAGCATG |  |  |
| afaD_F | GAAGCGCCTGACTGCCTTTTG | *afaD* gene detection | (Cruz-Córdova et al., 2020) |
| afaD_R | GATCACGTTGTAAGGCCGCC |  |  |
| papD_F | CACGCGAGTGATCTATCCGG | *papD* gene detection | (Cruz-Córdova et al., 2020) |
| papD_R | GTGATGAAGCGCACCTGGTC |  |  |
| hcp_F | GACGGCAACGCGATCAATTAC | *hcp* gene detection | (Cruz-Córdova et al., 2020) |
| hcp_R | GTTCTTGGTTGCACTCCACTG |  |  |
| gspD_F | GTCGACACCGATATCGGTGG | *gspD* gene detection | (Cruz-Córdova et al., 2020) |
| gspD_R | GGTAGACCACATGCAGGTTGC |  |  |
| virB_F | GCATCATGCAGAACGAGCTG | *virB* gene detection | (Cruz-Córdova et al., 2020) |
| virB_R | GACGGCTCGTACTTCTGCAC |  |  |
| tpsB_F | GTGGACATCGTGATGAAGCGC | *tpsB* gene detection | (Cruz-Córdova et al., 2020) |
| tpsB_R | CTTGCCGATGAAGTGACGGTG |  |  |
| stmpr1_F | GCCGCAGTGTTGGTTCGATCCA | *stmpr1*gene detection | (Alcaraz et al., 2021) |
| stmpr1_R | CAGTTCTCGGTGCACGGCTCTT |  |  |
| stmpr2_F | CGTGCCAGCTTCTCCAACTA | *stmpr2* gene detection | (Alcaraz et al., 2021) |
| stmpr2_R | AGGACTGTTGATGGTGCAGG |  |  |
| lip_F | CAGGCCTACAAGCTGCACTA | *lip* gene detection | (Al_Mosawy and AI_Muhanna, 2022) |
| lip_R | TTGACGAGGTCGATGGCATT |  |  |
| estr_F | CGGTGCCGAACTCGTAACCGG | *estr* gene detection | (ElBaradei and Yakout, 2022) |
| estr_R | CTTCCGGCCATGGCAGGCGAA |  |  |
| pls_F | ATCGACCTCGTCAAAGCCAG | *pls* gene detection | (Al_Mosawy and AI_Muhanna, 2022) |
| pls_R | AGGGTGGTCAGATAGGGGAC |  |  |
| feSR_F | CAATCGCAGCGTACCTACC | *feSR* gene detection | (Kalidasan et al., 2018) |
| feSR_R | CGGCCACGTTGAAGAACT |  |  |
| hemO/HO_F | CAGCAATTTCGCCCGTTTC | *hemO/HO* gene detection | (Kalidasan et al., 2018) |
| hemO/HO_R | GCTTGGCAGCCATCTTGTA |  |  |
| hyp1_F | GGCATCGTCGGCATCTT | *hyp1* gene detection | (Kalidasan et al., 2018) |
| hyp1_R | ACTTCACCCAGGCAATCG |  |  |
| hmuT_F | CATGCGCCACGACTGAT | *hmuT* gene detection | (Kalidasan et al., 2018) |
| hmuT_R | CATCACCCAGACCCGATTG |  |  |
| fur_F | TGACCGCCGAAGACATCTA | *fur* gene detection | (Kalidasan et al., 2018) |
| fur_R | GCGAGTGCTCTTCCAGTTC |  |  |
| spgM_F | CCKTGAAGTTGATCTCGCC | *spgM* gene detection | This work |
| spgM_R | GCTTCATCGAGGGCTAYTACC |  |  |
| rmlA_F | CGRCAACATCTTCCAYGG | *rmlA* gene detection | This work |
| rmlA_R | GGATGGTYTCGATGAARWTGG |  |  |
| smf1_F | GGAAGGTATGTCCGAGTCCG | *smf1*gene detection | (Nicoletti et al., 2011) |
| smf1_R | GCGGGTACGGCTACGATCAGTT |  |  |
| fliA_F | CGATCTCCTTCAGGTTCAGC | *fliA* gene detection | This work |
| fliA_R | CCAGGGSGCRTCGTTCGAG |  |  |
| rpfF_F | CTGGTCGACATCGTGGTG | *rpfF* gene detection | (Pompilio et al., 2011) |
| rpfF_R | TGATCCGCATCATTTCATGC |  |  |
| ax21_F | CCGCCAGGAAATCGACCACACC | *ax21* gene detection | This work |
| ax21_R | CGGTGTATTCCTTGTCGCCGTG |  |  |
| fliC-F | CGATCTCCGAGCGCTTCG | *fliC* gene detection | (Cruz-Córdova et al., 2020) |
| fliC-R | GAACAGCTGGCTGGAGAACG |  |  |
| pilU-F | CGACCACCATCGATTTCACTTCG | *pilU* gene detection | (Cruz-Córdova et al., 2020) |
| pilU-R | GACAGGTCCATCAGCAGCTG |  |  |
| IntI_F | GGGTCAAGGATCTGGATTTCG | *int1* gene detection | (Marathe et al., 2013) |
| IntI_R | ACATGCGTGTAAATCATCGTC |  |  |
| IntII_F | TTGCGAGTATCCATAACCTG | *int2* gene detection | (Abbasi et al., 2020) |
| IntII_R | TTACCTGCACTGGATTAAGC |  |  |
| Int1_F | GGCATCCAAGCAGCAAG | Class I integron gene cassette amplification | (Lévesque et al., 1995) |
| Int1_R | AAGCAGACTTGACCTGA |  |  |

Abbasi, E., Mondanizadeh, M., van Belkum, A., Ghaznavi-Rad, E., 2020. Multi-Drug-Resistant Diarrheagenic Escherichia coli Pathotypes in Pediatric Patients with Gastroenteritis from Central Iran. Infect. Drug Resist. 13, 1387–1396. https://doi.org/10.2147/IDR.S247732

Alcaraz, E., Centrón, D., Camicia, G., Quiroga, M.P., Di Conza, J., Passerini de Rossi, B., 2021. Stenotrophomonas maltophilia phenotypic and genotypic features through 4-year cystic fibrosis lung colonization. J. Med. Microbiol. 70. https://doi.org/10.1099/jmm.0.001281

Al_Mosawy, M.S., AI_Muhanna, A.S., 2022. PCR detection of Gene lip and plcN1 encoding lipase and lecithinase of Stenotrophomonas malltophilia isolated from different infection. Int. J. Health Sci. 6, 630–636. https://doi.org/10.53730/ijhs.v6nS9.12281

Baseri, Z., Dehghan, A., Yaghoubi, S., Razavi, Sh., 2021. Prevalence of resistance genes and antibiotic resistance profile among Stenotrophomonas maltophilia isolates from hospitalized patients in Iran. New Microbes New Infect. 44, 100943. https://doi.org/10.1016/j.nmni.2021.100943

Chen, S., Zhao, S., McDermott, P.F., Schroeder, C.M., White, D.G., Meng, J., 2005. A DNA microarray for identification of virulence and antimicrobial resistance genes in *Salmonella* serovars and *Escherichia coli*. Mol. Cell. Probes 19, 195–201. https://doi.org/10.1016/j.mcp.2004.11.008

Chen, S., Zhao, S., White, D.G., Schroeder, C.M., Lu, R., Yang, H., McDermott, P.F., Ayers, S., Meng, J., 2004. Characterization of Multiple-Antimicrobial-Resistant Salmonella Serovars Isolated from Retail Meats. Appl. Environ. Microbiol. 70, 1–7. https://doi.org/10.1128/AEM.70.1.1-7.2004

Cruz-Córdova, A., Mancilla-Rojano, J., Luna-Pineda, V.M., Escalona-Venegas, G., Cázares-Domínguez, V., Ormsby, C., Franco-Hernández, I., Zavala-Vega, S., Hernández, M.A., Medina-Pelcastre, M., Parra-Ortega, I., Rosa-Zamboni, D.D. la, Ochoa, S.A., Xicohtencatl-Cortes, J., 2020. Molecular Epidemiology, Antibiotic Resistance, and Virulence Traits of Stenotrophomonas maltophilia Strains Associated With an Outbreak in a Mexican Tertiary Care Hospital. Front. Cell. Infect. Microbiol. 10. https://doi.org/10.3389/fcimb.2020.00050

ElBaradei, A., Yakout, M.A., 2022. Stenotrophomonas maltophilia: Genotypic Characterization of Virulence Genes and The Effect of Ascorbic Acid on Biofilm Formation. Curr. Microbiol. 79, 180. https://doi.org/10.1007/s00284-022-02869-7

Fang, H., Ataker, F., Hedin, G., Dornbusch, K., 2008. Molecular epidemiology of extended-spectrum beta-lactamases among Escherichia coli isolates collected in a Swedish hospital and its associated health care facilities from 2001 to 2006. J. Clin. Microbiol. 46, 707–712. https://doi.org/10.1128/JCM.01943-07

Galimand, M., Guiyoule, A., Gerbaud, G., Rasoamanana, B., Chanteau, S., Carniel, E., Courvalin, P., 1997. Multidrug Resistance in Yersinia pestis Mediated by a Transferable Plasmid. N. Engl. J. Med. 337, 677–681. https://doi.org/10.1056/NEJM199709043371004

Helmy, O.M., Kashef, M.T., 2017. Different phenotypic and molecular mechanisms associated with multidrug resistance in Gram-negative clinical isolates from Egypt. Infect. Drug Resist. 10, 479–498. https://doi.org/10.2147/IDR.S147192

Kaczmarek, F.M., Dib-Hajj, F., Shang, W., Gootz, T.D., 2006. High-Level Carbapenem Resistance in a Klebsiella pneumoniae Clinical Isolate Is Due to the Combination of blaACT-1 β-Lactamase Production, Porin OmpK35/36 Insertional Inactivation, and Down-Regulation of the Phosphate Transport Porin PhoE. Antimicrob. Agents Chemother. 50, 3396–3406. https://doi.org/10.1128/AAC.00285-06

Kalidasan, V., Azman, A., Joseph, N., Kumar, S., Awang Hamat, R., Neela, V.K., 2018. Putative Iron Acquisition Systems in Stenotrophomonas maltophilia. Mol. J. Synth. Chem. Nat. Prod. Chem. 23, 2048. https://doi.org/10.3390/molecules23082048

Kim, S.H., Jeong, H.S., Kim, Y.H., Song, S.A., Lee, J.Y., Oh, S.H., Kim, H.R., Lee, J.N., Kho, W.-G., Shin, J.H., 2012. Evaluation of DNA Extraction Methods and Their Clinical Application for Direct Detection of Causative Bacteria in Continuous Ambulatory Peritoneal Dialysis Culture Fluids from Patients with Peritonitis by Using Broad-Range PCR. Ann. Lab. Med. 32, 119–125. https://doi.org/10.3343/alm.2012.32.2.119

Klimkaitė, L., Armalytė, J., Skerniškytė, J., Sužiedėlienė, E., 2020. The Toxin-Antitoxin Systems of the Opportunistic Pathogen Stenotrophomonas maltophilia of Environmental and Clinical Origin. Toxins 12, 635. https://doi.org/10.3390/toxins12100635

Klimkaitė, L., Ragaišis, I., Krasauskas, R., Ružauskas, M., Sužiedėlienė, E., Armalytė, J., 2023. Novel Antibiotic Resistance Genes Identified by Functional Gene Library Screening in Stenotrophomonas maltophilia and Chryseobacterium spp. Bacteria of Soil Origin. Int. J. Mol. Sci. 24, 6037. https://doi.org/10.3390/ijms24076037

Lastauskienė, E., Valskys, V., Stankevičiūtė, J., Kalcienė, V., Gėgžna, V., Kavoliūnas, J., Ružauskas, M., Armalytė, J., 2021. The Impact of Intensive Fish Farming on Pond Sediment Microbiome and Antibiotic Resistance Gene Composition. Front. Vet. Sci. 8, 673756. https://doi.org/10.3389/fvets.2021.673756

Lévesque, C., Piché, L., Larose, C., Roy, P.H., 1995. PCR mapping of integrons reveals several novel combinations of resistance genes. Antimicrob. Agents Chemother. 39, 185–191. https://doi.org/10.1128/AAC.39.1.185

Lira, F., Berg, G., Martínez, J.L., 2017. Double-Face Meets the Bacterial World: The Opportunistic Pathogen Stenotrophomonas maltophilia. Front. Microbiol. 8.

Lira, F., Hernández, A., Belda, E., Sánchez, M.B., Moya, A., Silva, F.J., Martínez, J.L., 2012. Whole-Genome Sequence of Stenotrophomonas maltophilia D457, a Clinical Isolate and a Model Strain. J. Bacteriol. 194, 3563–3564. https://doi.org/10.1128/JB.00602-12

Malekan, M., Tabaraie, B., Akhoundtabar, L., Afrough, P., Behrouzi, A., 2017. Distribution of Class I Integron and smqnr Resistance Gene Among Stenotrophomonas maltophilia Isolated from Clinical Samples in Iran. Avicenna J. Med. Biotechnol. 9, 138–141.

Marathe, N.P., Regina, V.R., Walujkar, S.A., Charan, S.S., Moore, E.R.B., Larsson, D.G.J., Shouche, Y.S., 2013. A Treatment Plant Receiving Waste Water from Multiple Bulk Drug Manufacturers Is a Reservoir for Highly Multi-Drug Resistant Integron-Bearing Bacteria. PLOS ONE 8, e77310. https://doi.org/10.1371/journal.pone.0077310

Nicoletti, M., Iacobino, A., Prosseda, G., Fiscarelli, E., Zarrilli, R., De Carolis, E., Petrucca, A., Nencioni, L., Colonna, B., Casalino, M., 2011. Stenotrophomonas maltophilia strains from cystic fibrosis patients: genomic variability and molecular characterization of some virulence determinants. Int. J. Med. Microbiol. IJMM 301, 34–43. https://doi.org/10.1016/j.ijmm.2010.07.003

Pompilio, A., Pomponio, S., Crocetta, V., Gherardi, G., Verginelli, F., Fiscarelli, E., Dicuonzo, G., Savini, V., D’Antonio, D., Di Bonaventura, G., 2011. Phenotypic and genotypic characterization of Stenotrophomonas maltophiliaisolates from patients with cystic fibrosis: Genome diversity, biofilm formation, and virulence. BMC Microbiol. 11, 159. https://doi.org/10.1186/1471-2180-11-159

Saleh, R.O., Hussen, B.M., Mubarak, S.M.H., Mostafavi, S.K.S., 2021. High diversity of virulent and multidrug-resistant Stenotrophomonas maltophilia in Iraq. Gene Rep. 23, 101124. https://doi.org/10.1016/j.genrep.2021.101124

Versalovic, J., Schneider, M., Bruijn, F.J.D., Lupski, J.R., 1994. Genomic fingerprinting of bacteria using repetitive sequence-based polymerase chain reaction. Methods Mol. Cell. Biol. 5, 25–40.

Whitby, P.W., Carter, K.B., Burns, J.L., Royall, J.A., LiPuma, J.J., Stull, T.L., 2000. Identification and detection of Stenotrophomonas maltophilia by rRNA-directed PCR. J. Clin. Microbiol. 38, 4305–4309. https://doi.org/10.1128/JCM.38.12.4305-4309.2000

Yu, J.R., Chung, J.S., Chai, J.Y., 1997. Different RAPD patterns between Metagonimus yokogawai and Metagonimus Miyata type. Korean J. Parasitol. 35, 295–298. https://doi.org/10.3347/kjp.1997.35.4.295
